# The Three-Species Consortium of Genetically Improved Strains *Cupriavidus necator* RW112, *Burkholderia xenovorans* RW118, and *Pseudomonas pseudoalcaligenes* RW120 Grows with Technical Polychlorobiphenyl, Aroclor 1242

**DOI:** 10.3389/fmicb.2013.00090

**Published:** 2013-04-29

**Authors:** Verónica Hernández-Sánchez, Elke Lang, Regina-Michaela Wittich

**Affiliations:** ^1^Department of Environmental Protection, Experimental Station of the Zaidín, Spanish High Council for Scientific ResearchGranada, Spain; ^2^Leibniz Institute – German Collection of Microorganisms and Cell CulturesBraunschweig, Germany

**Keywords:** *Cupriavidus necator* RW112, *Burkholderia xenovorans* RW118, *Pseudomonas pseudoalcaligenes* RW120, genetic engineering, polychlorinated biphenyls, Aroclor 1242

## Abstract

*Burkholderia xenovorans* LB400, *Cupriavidus necator* H850, and *Pseudomonas pseudoalcaligenes* KF707 are bacterial strains able to mineralize biphenyl and to co-oxidize many of its halogenated derivatives (PCBs). Only strain LB400 also mineralizes a few mono- and dichlorobiphenyls, due to the presence of a functioning chlorocatechol pathway. Here, we used a Tn*5*-based minitransposon shuttle system to chromosomically introduce genes *tcbRCDEF*, encoding the chlorocatechol pathway into KF707, and genes *cbdABC* encoding a 2-chlorobenzoate 1,2-dioxygenase into KF707 and LB400, as well as transposon Tn*4653* from the TOL plasmid, providing genes *xylXYZL*, encoding a broad-range toluate (methylbenzoate) dioxygenase and its dihydrodiol dehydrogenase, to extend the range for the mineralization of halogenated benzoates in LB400 and in KF707 through co-oxidation of halobenzoates into chlorocatechols. The engineered derivatives of LB400 and KF707 thus gained the ability for the mineralization of all isomeric monochloro- and bromobenzoates of the so-called lower pathway which, consequently, also allowed the mineralization of all monochlorobiphenyls and a number of di- and trichlorobiphenyls, thus preventing the accumulation of halobenzoates and of catabolites thereof. LB400 and KF707 also grow with the two commercial PCB formulations, Aroclor 1221 and Aroclor 1232, as the sole carbon and energy sources, but not with higher halogenated PCB mixtures, similar to the already published strain RW112. Repeated exposition of the modified LB400 to short pulses of UV light, over a prolonged period of time, allowed the isolation of a derivative of LB400, termed RW118, capable of growth with Aroclor 1016 still containing only traces of biphenyl, and in co-culture with modified KF707 termed RW120, and modified H850 (RW112) with Aroclor 1242, the commercial mixture already void of biphenyl and monochlorobiphenyls.

## Introduction

PCBs represent poorly water-soluble, highly persistent, and toxic halo-organic compounds in the biosphere (Harris et al., 1993; Carpenter, 1998). The technical mixtures of mid and highly halogenated PCBs are rather recalcitrant toward bacterial degradation and consequently have accumulated in the ecosphere. Their congeners are subject of anaerobic bacterial dehalogenation (Tiedje et al., 1993) which proceeds slowly under strictly anoxic environmental conditions due to the overall low energy yield of these reactions although they are thermodynamically favorable, whilst aerobic bacteria can use the entire carbon skeleton of (halo-) biphenyl for efficient growth. Highly halogenated PCBs thus yield less chlorinated congeners in anoxic environments which, upon migration in soil or sediments to more oxic zones then can be attacked by enzyme systems of aerobic bacteria. The aerobic mineralization of oligo- or polyhalogenated aromatics in general is a typically more rapid bioprocess, due to the much higher yield of energy (Abramowicz, 1990; Sander et al., 1991; Higson, 1992; Potrawfke et al., 1998b) from carbon. The literature published on the biodegradation of PCBs, including general, biochemical, genetic, and applied aspects, has been reviewed only a few years ago (Field and Sierra-Alvarez, 2008; Furukawa and Fujihara, 2008; Pieper and Seeger, 2008).

Publications from the past 20 years reported that only a very few dichlorinated congeners mineralizing bacteria could be isolated and partially characterized, or obtained upon natural, horizontal genetic exchange between biphenyl and chlorobenzoates mineralizing species (Mokross et al., 1990; Havel and Reineke, 1991; Adams et al., 1992; Hickey et al., 1992; Kim and Picardal, 2001; Adebusoye et al., 2008), or by introduction of plasmid-based catabolic genes (Havel and Reineke, 1993; Reineke, 1998; Hrywna et al., 1999; Rodrigues et al., 2001). Nevertheless, none of these strains could grow on technical mixtures of PCBs, except only a few on Aroclor 1221 which contains about 10% of biphenyl, 63% of mono, and 27% of dichlorinated biphenyls, apart from traces of trichlorinated congeners.

Here, we report the construction of two recombinant bacterial strain, based on *Burkholderia xenovorans* LB400, then termed RW118, and on *Pseudomonas pseudoalcaligenes* KF707, then termed RW120, upon genetic manipulations and exposure to UV as a potential mutagenic agent, and finally capable of good growth together with the earlier constructed strain RW112 based on *Cupriavidus necator* H850, on Aroclor 1242, containing 44% of chlorine per molecule, as the sole source of carbon and energy.

## Materials and Methods

### Bacterial strains and growth conditions

Strains *Burkholderia xenovorans* LB400 (DSM 17367) and *Pseudomonas pseudoalcaligenes* KF707 (DSM 10086) were obtained from the Leibniz-Institut DSMZ – Deutsche Sammlung von Mikroorganismen und Zellkulturen, Braunschweig, Germany. Strain *Cupriavidus necator* RW112 (DSM 13439) had been constructed earlier in a similar manner (Wittich and Wolff, 2007). *Escherichia coli* strains were grown in Luria Bertani (LB) medium and appropriate antibiotics. All other strains were grown in mineral salts solution under standard conditions (Bopp, 1986; Sander et al., 1991) with benzoate or biphenyl at pH 7.2 and 28°C. The initial pH was raised to 7.5 when growing on chlorobenzoates or chlorobiphenyls (5 mM), and to initially pH 8.0 when growing it on Aroclor 1232, 1016, or 1242, respectively, to compensate for acidification of the culture medium due to the release of protons, and to avoid phosphate buffer concentrations above 27 mM which inhibited growth. 20 ml cultures with derivatives of LB400 and individual chlorobiphenyls or PCB mixtures were grown in 100 ml, PTFE-sealed glass Erlenmeyer flasks on an overhead spinner between 10 and 50 rpm. At time points depicted in the figures, flasks were removed and 10 μl taken for the determination of living cells colony forming units (CFU) by spreading aliquots of appropriate serial dilutions on solid LB medium. Colonies were counted after two or more days of incubation at 28°C. At the same time points separate 1.0 ml aliquots were taken for analytical purposes [high-performance liquid chromatography (HPLC), OD_600_, and chloride concentration]. Agar Noble (Difco) was used for the preparation of solid mineral salts medium.

*E. coli* donor strain CC118 λ pir harboring the delivery vector on a suicide plasmid (Tn5-based delivery plasmid, Ap^r^ Km^r^ pUTkm, de Lorenzo and Timmis, 1994), with the catabolic inserts on pBJ44 or pAL1 (Jakobs, 1998; Lehning, 1998), respectively, and *E. coli* helper strain HB101 with pRK600 (Cm^r^ ColE1 RK2-Mob^+^ RK2-Tra^+^ derivative of pRK2013, Figurski and Helinski, 1979) for the tri-parental mating were grown in LB medium with the appropriate antibiotics, kanamycin, and ampicillin, at 70 μg ml^−1^ at 37°C. The plasmids pBJ44 and pAL1 were obtained from B. Jakobs and A. Lehning, respectively [Helmholtz-Center for Infection Research (former German Research Centre for Biotechnology), Braunschweig, Germany]. pBJ44 harbors genes encoding the entire chlorocatechol pathway (Klemba et al., 2000), and pAL1 harbors genes *cbdABC* (positions 288–3221, GenBank accession no. X79076) encoding a 2-halobenzoate dioxygenase from the 2-chlorobenzoate degrading strain *Pseudomonas cepacia* 2CBS (Haak et al., 1995). *Pseudomonas putida* mt-2 was the donor of genes *xylXYZL* (positions 463–4266, GenBank accession no. M64747), found on transposon Tn*4653* of the TOL plasmid pWW0 (Williams and Murray, 1974; Greated et al., 2002). Strain mt-2 was grown at 28°C on 5 mM 3-methylbenzoate. Bacterial growth of donor and recipient strains was determined by measuring Optical Density (OD) of 1 ml samples or appropriate dilutions of the culture medium at E_600_ in a Shimadzu UV-2100 spectrophotometer, or by enumeration of CFU of generated hybrid strains in experiments with PCBs. Strains and their special characteristics are listed in Table 1.

**Table 1:** **Strains used in this work, and new substrates mineralized (chlorobenzoates and chlorobiphenyls)**.

Strains and their plasmids	Characteristics, and/or mineralization of	Source
*Escherichia coli* CC118 (λ pir)::pBJ44	Chlorocatechol pathway cassette	Klemba et al. (2000)
*Escherichia coli* CC118 (λ pir)::pAL1	2-Halobenzoate 1,2-dioxygenase cassette	Lehning (1998)
*Escherichia coli* HB101::pRK600	RK2-based Tra- and Mob-functions (helper strain)	Kessler et al. (1992)
*Burkholderia xenovorans* LB400	Benzoate, 3-chlorobenzoate, biphenyl, 3-chlorobiphenyl, 2,3′-dichlorobiphenyl	Bopp (1986)
*Pseudomonas pseudoalcaligenes* KF707	Benzoate, biphenyl	Furukawa and Miyazaki (1986)
*Pseudomonas putida* mt-2 pTOL (Tn4653)	Methylbenzoates (broad-spectrum alkylbenzoate 1,2-dioxygenase)	Williams and Murray (1974)
*Cupriavidus necator* RW112	Benzoate, all monochlorobenzoates, 3,5-dichlorobenzoate, biphenyl, all monochlorobiphenyls, 3,5-dichloro-, 2,2′-dichloro-, 2,3′-dichloro, and 2,4′-dichlorobiphenyl	Wittich and Wolff (2007)
*Burkholderia xenovorans* RW118	Benzoate, all monochlorobenzoates, 3,5-dichlorobenzoate, biphenyl, all monochlorobiphenyls, 3,5-dichloro-, 2,2′-dichloro-, 2,3′-dichloro-, and 2,4′-dichlorobiphenyl	This study
*Pseudomonas pseudoalcaligenes* RW120	Benzoate, all monochlorobenzoates, 3,5-dichlorobenzoate, biphenyl, all monochlorobiphenyls, 3,5-dichloro-, 2,2′-dichloro-, 2,3′-dichloro-, and 2,4′-dichlorobiphenyl	This study

### Chemicals

Biphenyl and chlorobiphenyl congeners were from Sigma-Aldrich, Steinheim, Germany, and Cerilliant Co., Round Rock, TX, USA. Aroclor mixtures were from Promochem, Wesel, Germany (Aroclor 1232, Lot N32SI, Aroclor 1016, Lot W12507); and ULTRA Scientific, Kingstown, RI, USA (Aroclor 1221, Lot NT01017; Aroclor 1242, Lot NT010020; Aroclor 1248, Lot NT01582). 4-bromobiphenyl was from Alfa Aesar, Karlsruhe, Germany. 4,4′-Dichlorobiphenyl (Sigma-Aldrich) was purified before use from three polar contaminants on preparative TLC plates with silica gel as the solid, and hexane as the mobile phase. All other chemicals were of the highest purity commercially available.

### Generation of hybrid strains

For mating experiments a standard protocol was used (de Lorenzo and Timmis, 1994). Recipient strains were mixed on a filter disk with the donor strain, *E. coli* CC118 λ pir, carrying the respective catabolic insert on a suicide plasmid, and the *E. coli* HB101 helper strain in a 1:1:1 ratio and incubated on solid LB medium in the absence of antibiotics overnight. The filter disk was then placed in a test tube and cells separated from the disk upon addition of 1 ml of 1% saline and whirl mixing. One hundred micro liters of this solution were plated directly onto solid mineral salts medium containing the target carbon source, 2-chlorobenzoate in the case of transfer of *cbdABC*, and 3-chlorobenzoate in the case of transfer of catabolic genes *tcbRCDEF*. Colonies appearing after about 3–4 weeks were subcultured on the same medium, then purified by passage on solid LB medium and retransfer to solid selective medium. For the transfer of the transposon from the TOL plasmid with the catabolic genes *xylXYZL* the recipient strain was incubated overnight with *Pseudomonas putida* mt-2 on LB plates and processed as before, selecting for additional growth on 4-chlorobenzoate. The obtained derivative of KF707 was termed RW120. Exposure to UV pulses of 1 s was realized inside of a clean bench using the internal UV system at a distance of 60 cm. The strain resulting from already modified LB400 was termed RW118.

### Determination of oxygen uptake

Specific rates of oxygen uptake by washed cell suspensions (resting cells) were determined polarographically with the oxygen electrode DW-1 system from Hansatech Instruments, King’s Lynn, Norfolk, UK.

### DNA procedures

For the preparation of genomic DNA, the Wizard Genomic DNA Purification Kit from Promega (Madison, WI, USA) was used. For the amplification of DNA fragments, Takara ExTaq, or PrimeStar DNA polymerase (TAKARA BIO INC., Shiga, Japan) were used under the standard protocols supplied by the manufacturer. The following oligonucleotides were used: RW7 (5′-TAACTAAGCGCTGCCTTT-3′) and RW8 (5′-ATTGCTTGGGAAAACAAT-3′) for amplification of the *tcbRCDEF* operon for the mineralization of halocatechols, VH1 (5′-TGCAGTGTCCGGTTTGATAG-3′) and VH4 (5′-GAAAAT GAGCCTCAGGGGTC-3′) for the gene encoding the toluate dioxygenase and the correlated dihydrodiol dehydrogenase, RW11 (5′-TCTCCGCAGTCTATAAGGCT-3′) and RW12 (5′-ACGCTTCGCTGCCTTATTCG-3′) for the gene coding for the 2-halobenzoate dioxygenase system.

### Analytical techniques

PCB mixtures (residual amounts) were extracted from the culture media with hexane and analyzed by HPLC and/or gas chromatography (GC). HPLC with UV-DAD detection was performed on an Agilent 1050 liquid chromatograph system to determine the residual concentration of PCB mixtures. Separation of the entire PCB fraction from polar compounds was done on a 3.9 mm × 150 mm Nova-Pak C18 – 4 μm cartridge column as the solid phase and detection at 210 nm; the liquid phase was 80% acetonitrile in water (vol:vol) at pH 1.5 (phosphoric acid, 0.1%). GC was performed with simultaneous mass and ECD detection, in order to include non-halogenated biphenyl, and was applied to determine the concentrations of individual congeners. Extracted compound mixtures were analyzed on an Agilent 6890 gas chromatograph coupled to an ECD and 5972 mass detector via a splitter. Separation was on a 50 mm × 0.2 mm Hewlett Packard Ultra 2 capillary column and hydrogen as the carrier. The injector temperature was 250°C. The temperature program started with 80°C for 3 min, followed by a ramp to 290°C at 6°C/min, and the final temperature was kept constant for additional 20 min. Peaks generated by either the ECD or the mass detector were identified by comparison with commercial PCB standards dissolved in cyclohexane (Dr. Ehrenstorfer GmbH, Augsburg, Germany), and literature data (Frame et al., 1996) which also served for the correction of incompletely dissolved signals. Chloride ion concentrations were determined by use of a chloride-selective electrode, model Cl-6732 from PASCO scientific, Roseville, CA, USA.

## Results and Discussion

### Construction of derivatives of LB400 and KF707

*Burkholderia xenovorans* LB400, already carrying a catabolic sequence for the mineralization of chlorocatechols, and *Pseudomonas pseudoalcaligenes* KF707 were subjected to tri-parental mating procedures upon having been pregrown on biphenyl. From a mating with *E. coli* CC118 λ pir carrying genes *cbdABC* within the suicide plasmid pAL1, coding for a 2-chlorobenzoate dioxygenase, and *E. coli* HB101 carrying the helper plasmid pRK600, the selection of positive clones was performed on this substrate. Colonies appeared on mineral salts agar with this novel target substrate after around 3 weeks, and were purified from contaminating *E. coli* cells on the same solid medium. This derivative of LB400 was termed RW116 and further subjected to a mating with *Pseudomonas putida* mt-2 for transfer of transposon Tn*4653* from the TOL plasmid, hosting genes *xylXYZL* of the TOL lower pathway for the mineralization of methyl benzoates by the broad substrate range toluate 1,2-dioxygenase and for the corresponding dihydrodiol dehydrogenase for aromatization to the corresponding halocatechols under decarboxylation. This toluate dioxygenase is capable of co-oxidation of isomeric halobenzoates into the corresponding halocatechols. Positive clones were found upon incubation of the washed mating mix on solid mineral salts agar with 4-chlorobenzoate as the substrate after at least 2 weeks of incubation, and termed RW117. This strain grew with all isomers of monochlorobenzoates and 4-bromobenzoate, but not with dichlorobenzoates, probably due to the narrow substrate range of the transport system for benzoates carrying bulky substituents. Chlorocatechols mineralized were 3-chloro-, 4-chloro-, 4-bromo, and 3,5-dichlorocatechol; 4,5-dichloro- and tetrachlorocatechol did not serve for growth. The same procedure was applied for the modification of KF707, with an identical outcome, finally yielding strain RW120.

Chlorobiphenyls being mineralized were 3-chloro- and 2,3′-dichlorobiphenyl, as before by LB400, but now also 2-chloro- and 4-chlorobiphenyl, as well as 2,2′-dichloro-, 2,4′-dichloro-, and 3,5-dichlorobiphenyl (with regard to the latter, 3,5-dichlorobenzoate was not detectable by HPLC in the spent culture medium). 4,4′-Dichlorobiphenyl, astonishingly, did serve as a carbon- and energy source for RW117, but for a number of generations of growth only. It had been shown that LB400 could attack this structure (Bopp, 1986), and that the gene product of *bphK* of LB400, a glutathione *S*-transferase, can reductively dehalogenate correspondingly halogenated HOPDAs (chlorinated 2-hydroxy-6-oxo-6-phenyl-2,4-dienoates), obtained upon 2,3-dioxygenation of (chlorinated) biphenyl and subsequent *meta*-cleavage (Seah et al., 2000; Fortin et al., 2006; Tocheva et al., 2006). We have to assume some negative interaction with regard to the constitutive induction of the catabolic pathway(s). Although LB400 already showed some growth on Aroclor 1221, its derivative, RW117, could also grow well on Aroclor 1232 (data not shown), similar to the genetically improved derivative of *Cupriavidus necator* H850, strain RW112 (Wittich and Wolff, 2007). On higher halogenated PCB mixtures such as Aroclor 1016 and 1242, RW117 survived for a couple of days but did not show any significant growth. With RW120 almost identical results were obtained.

**Figure 1:**
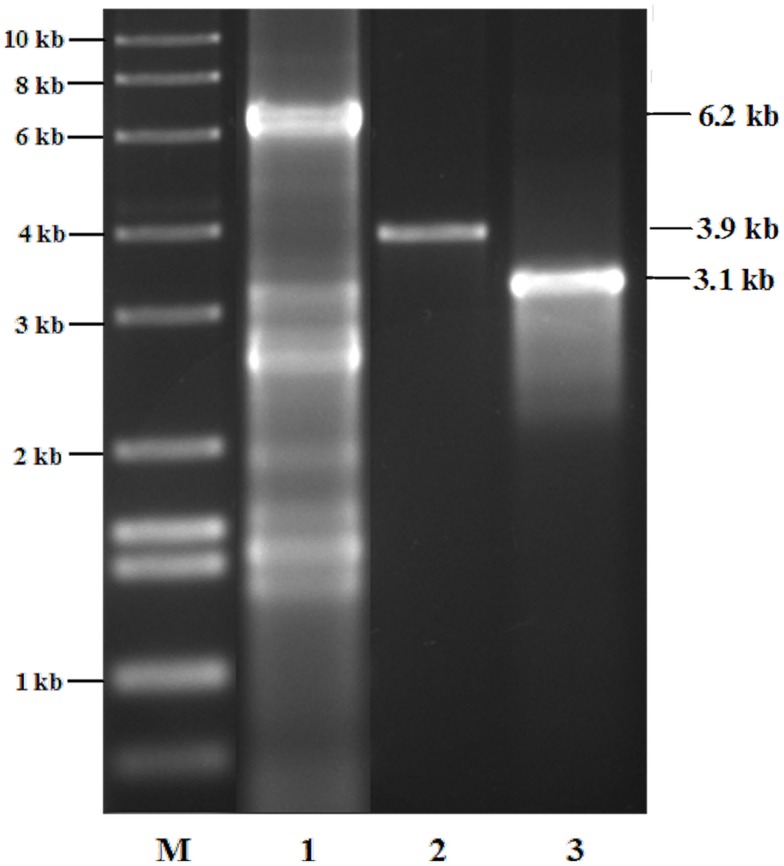
**Agarose gel electrophoretogram of DNA fragments corresponding to catabolic genes of the autochthonous chlorocatechol pathway of LB400, and/or to those introduced via transposon structures, of genes encoding the 2-halobenzoate 1,2-dioxygenase, and a toluate dioxygenase of broad substrate range for the co-oxidation of halocatechols in its derivative RW118, as well as in RW112 and RW120: the latter two strains give an identical pattern (not shown), refer to Figure 2 for details**.

**Figure 2:**
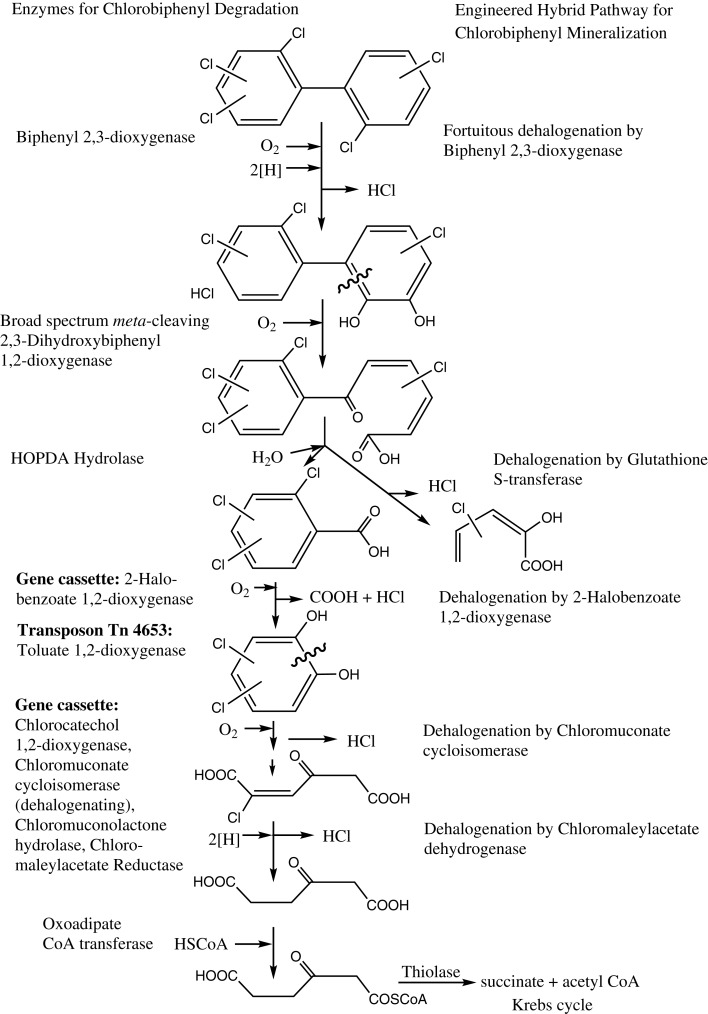
**Proposed catabolic pathways for the mineralization of halogenated biphenyls by the engineered bacterial strains**. The metabolism of PCBs by all strains proceeds through a number of dehalogenating reactions.

In order to demonstrate the chromosomal insertion of the above-mentioned sequences, as well as the presence of the chlorocatechol pathway of LB400, we amplified the corresponding fragments from the two chromosomes or megaplasmids of this strain, and in a similar manner, from the former constructed RW112 and the of strain RW120 constructed in this study. A preparation of genomic DNA of RW112, RW117, and RW120, upon growth on 4-chlorobenzoate, served as the template for the PCR reactions. Results (Figure 1) demonstrate fragments of the exact expected length amplified from the respective genes, irrespectively of already harbored in LB400, or of newly introduced ones. The fragment in lane 1 corresponds to the gene sequence of the chlorocatechol pathway, the amplification yielded a corresponding fragment of 6.2 kb (exact length, 6.206 bp). This primer pair had originally been designed for the amplification of genes *tcbRCDEF* of plasmid p51 (van der Meer et al., 1991) which may explain the strong background in LB400. Amplification of genes *cbdABC* encoding the 2-halobenzoate 1,2-dioxygenase, and of genes *xylXYZL* encoding the *meta*-toluate 1,2-dioxygenase system and the corresponding dihydrodiol dehydrogenase) gave clear bands of about 3.1 kb (lane 2, exact length, 3.163 bp), and 3.9 kb (lane 3, exact length, 3.900 bp), so far as expected; amplifications from RW112 and RW120 genomic DNA yielded exactly the same pattern (not shown). A catabolic scheme (Figure 2) shows the reaction sequences for the breakdown of PCBs and the gene products, although the chlorocatechol pathway of LB400 has not been investigated in detail, but based on the expected length of the fragment obtained, one can assume an almost identical sequence and organization of this catabolic pathway (Potrawfke et al., 1998a), as well as horizontal gene transfer between a number of bacterial species since we identified a similar one also in the 1,2,3,4-tetrachlorobenzene mineralizing *Pseudomonas chlororaphis* RW71 (Potrawfke et al., 1998b, data not shown).

### Further development of strains

In a number of previous publications, the constitutive expression of the entire catabolic pathway for the mineralization of biphenyl had been reported, and a positive regulator, BphR1 (former ORF0, Erickson and Mondello, 1992; Beltrametti et al., 2001) identified (Denef et al., 2004), and it was shown, that this gene product represents a mediator for the promoter of *bphA1*. Because such regulatory elements could be of some importance for the further development of LB400 and its derivatives, especially toward potential growth on PCB mixtures which do not contain biphenyl or monochlorobiphenyls any more, the induction state, as well as the capacity for co-oxidation of PCBs requires to be validated. One reason is a relative recent publication (Parnell et al., 2010) which reported that the carbon source and growth phase could have some effects on the expression of the catabolic *bph* operon, especially could influence the catabolic gene expression and related enzyme activity for the productive breakdown of the PCBs. These authors argued, from their results obtained from quantitative reverse transcription PCR which showed expression of the catabolic genes in the presence of succinate and PCB mixtures, but the absence of catabolic turnover for PCBs during a so-called transition phase leading into the stationary phase. Our results from the determination of relative oxygen uptake rates of cells grown with succinate, succinate with biphenyl, and with succinate in the presence of minute amounts of Aroclor 1242, showed very clearly, that no more turnover of PCBs occurred when cells got void of succinate as the source of energy. However, the oxidation of biphenyl started again, after 5 h of being void of succinate, about 1 h after having supplied succinate again to starved cells. The simple explanation for the findings of these authors is that their LB400 cells had no more or not sufficient reduction equivalents left for the (co-) oxidation of PCBs. However, when PCB mixtures could be partially mineralized and serve for growth under production of energy and generate carbon for the anabolism, no heavy detrimental effects should be expected. Since RW117 could already grow well with Aroclor 1232, similar to RW112 (Wittich and Wolff, 2007), we argued that further mutagenization and the presence of selective pressure could improve its capacity for growth on higher halogenated mixtures. Cells growing in mineral salts medium with 4-bromobiphenyl and traces of Aroclors 1016 and 1242 were subjected to short pulses of UV light which killed about 90–95% of the population. Aliquots were then added to another flask with salts medium but with Aroclor 1016, as well as Aroclor 1242, as the target carbon and energy source. Although this simple approach by using UV as a mutagen may appear a bit old-fashioned in an era of much more rational molecular techniques as side specific mutagenesis, gene shuffling, etc., after about 5 months we got some growth with Aroclor 1016, and some time later, with Aroclor 1242. From this culture, RW118 was isolated and used for further experiments.

### Growth of the consortium of RW112, RW118, and RW120 with AROCLOR 1242

**Figure 3:**
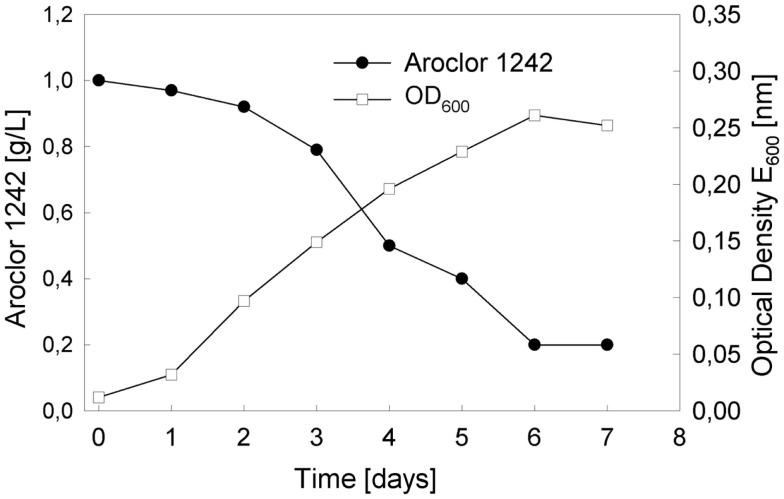
**Growth of the genetically engineered three-species culture as indicated by the depletion of the sum of congeners over time and the increase in biomass, as determined by measuring Optical Density (OD_600_) of the culture broth**.

As mentioned above, our three GMOs can only grow with Aroclor 1232 as a carbon source. However, in a preliminary experiment with a mixed consortium of all strains, we observed growth, first on Aroclor 1016, and by using this culture as an inoculum, also on Aroclor 1242. For the following experiment, strains RW112, RW118, and RW120 were inoculated from solid medium with 4-bromobiphenyl as a cheap carbon source and maintenance medium (4-chlorobiphenyl is only available at analytical amounts) into 20 ml of fresh mineral salts medium. Cells were grown at a concentration of 1 g/l of Aroclor 1242 directly added to the sterile medium without previous sterilization of this PCB mixture. When growth of the culture slowed significantly down and cells became fully immotile upon microscopic investigation, they were transferred to fresh medium with the same carbon source. During growth, the medium became turbid as expected, but did not change in its color, indicating that no yellow chloro-HOPDAs or other, brownish or black oxidized or polymerized toxic catabolites accumulated, which could exert detrimental effects toward the cells of the consortium. Such toxic effect by Aroclor 1242 on the parent strain of RW118, LB400, was described earlier (Parnell et al., 2006). The growth curve (Figure 3) demonstrates that the depletion of Aroclor 1242 is almost linear, as well as the increase in cell mass over time. In order to determine the relative composition of the three strains, aliquots were diluted appropriately and plated on solid LB medium, since the color and appearance of individual colonies were sufficiently different from each other. Over time, RW120 dominated slightly over RW112 and RW118. A riboprint analysis (Bruce, 1996) clearly confirmed the pattern of *Pseudomonas pseudoalcaligenes* (RW120) when re-isolated from the culture growing with Aroclor 1242.

Residual Aroclor 1242 congeners were extracted with hexane from the culture medium when growth of the consortium came to a still stand, the organic phase separated, dried with anhydrous sodium sulfate, and analyzed by GC-MS-ECD. For comparison with the initial concentrations of each congener, the last significant peak of the chromatogram, a mixture of 2,3,4,3′,4′-pentachloro-, and 2,3,4,2′,3′,6′-hexachlorobiphenyl, representing peak 40, was used as the internal standard. The data obtained demonstrate a high rate of depletion of the majority of the congeners (Table 2) which may correlate with the significant growth yield as determined by OD; however, we did not analyze the spent culture medium for accumulated metabolites (due to the actually high costs for Aroclor standards and a relatively high amount needed for HPLC-MS analyses). Especially chlorobenzoates had been identified in various previous publications, although at least the monohalogenated isomers/congeners, and probably some dihalogenated ones, can be mineralized by RW112, RW118, and RW120. The substrate specificity of the necessary transporters may limit the transfer of chlorobenzoates into the cell (Yuroff et al., 2003), although such structures may be mineralized when delivered intracellularly from PCB congeners. The main reason for the efficient degradation of Aroclor 1242 in co-culture by the three strains, however, may be found in their different substrate specificities (Gibson et al., 1993; Williams et al., 1997).

**Table 2:** **Depletion of individual congeners of Aroclor 1242 by growing cells of *Cupriavidus necator* RW112, *Burkholderia xenovorans* RW118, and *Pseudomonas pseudoalcaligenes* RW120**.

Peak no.	Congener(s)	Weight %	Depletion %
1	2,2′/2,6	2.7	100
2	2,4/2,5	0.9	100
3	2,3′	1.2	100
4	2,3/2,4′	5.8	100
5	2,6,2′	0.9	100
6	2,5,2′	8.7	100
7	2,4,2′/4,4′	4.3	100
8	2,3,6/2,6,3′	0.7	100
9	2,3,2′/2,6,4′	4.8	100
10	2,5,3′	1.1	100
11	2,4,3′	7.0	100
12	2,5,4′	7.0	100
13	2,4,4′	6.5	100
14	2′,3,4/2,5,2′,6′	3.3	100
15	2,3,4′/2,4,2′,6′	0.9	100
16	2,3,6,2′	0.5	100
17	2,3,2′′,6′	0.5	100
18	2,5,2′,5′	3.1	100
19	2,4,2′,5′/2,3,5,2′	2.6	100
20	2,4,2′,4′	1.1	086
21	2,4,5,2′	1.4	090
22	2,3,2′,5′	3.4	100
23	3,4,4′/2,3,2′,4′	3.7	081
24	2,3,4,2′/2,3,6,4′/2,6,3′4′	3.0	086
25	2,3,2′,3′	0.9	100
26	2,4,5,4′	1.9	068
27	2,5,3′,4′	3.9	100
28	2,4,3′,4′/2,3,6,2′,5′	4.8	091
29	2,3,6,2′,4′	1.3	078
30	2,3,3′,4′/2,3,4,4′	2.9	067
31	2,3,6,2′,3′/2,3,5,2′,5′	1.0	089
32	2,3,5,2′,4′/2,4,5,2′,5′	1.1	100
33	2,4,5,2′,4′	1.0	094
34	2,4,5,2′,3′/2,3,5,6,2′,6′	1.0	062
35	2,3,4,2′,5′	1.0	073
36	2,3,4,2′,4′	0.7	052
37	2,3,6,3′,4′/3,4,3′,4′	1.1	030
38	2,3,4,2′,3′	0.7	032
39	2,3,6,2′,4′,5′/2,4,5,3′,4′	0.7	029
40	**2,3,4,3**′**,4**′**/2,3,4,2**′**,3**′**,6**′	1.0	0

*Bold: used as internal standard*.

## Conclusion

We highlighted in this study the feasibility of a defined three-species consortium of genetically modified *Cupriavidus necator* H850, *Burkholderia xenovorans* LB400, and of *Pseudomonas pseudoalcaligenes* KF707 to be genetically manipulated, and gain an initially unexpected potential for co-culture growth on the technical Aroclor 1242 mixture in the absence of other carbon sources. Strain RW118 has also been sent out for high-throughput sequencing (Japan) of its genome, in order to compare its sequence with that of the original LB400, and screen it for modifications which may have occurred over time during the process of modification and adaptation. An in depth genetic analysis may also follow on the rough data already obtained by this procedure from modified H850 and KF707.

## Conflict of Interest Statement

The authors declare that the research was conducted in the absence of any commercial or financial relationships that could be construed as a potential conflict of interest.
